# [Retracted] Hsp90 inhibitor 17-AAG inhibits stem cell-like properties and chemoresistance in osteosarcoma cells via the Hedgehog signaling pathway

**DOI:** 10.3892/or.2026.9137

**Published:** 2026-05-18

**Authors:** Xiong Shu, Huiqi Liu, Rui Zhen, Yongsheng Jie, Lei Chen, Hui Qi, Chao Wang, Renxian Wang, Dafu Chen, Yuliang Ran

Oncol Rep 44: 313–324, 2020; DOI: 10.3892/or.2020.7597

Following the publication of the above article and a corrigendum (doi: 10.3892/or.2023.8593) that was published 3 years ago to resolve the issue of duplicated data that had been noted comparing Figs. 1B and C, and 3B and C, respectively, a concerned reader has contacted the Editorial Office to draw our attention to the fact that a series of potential duplications of western blot data had also been noted comparing Fig. 4 with Fig. 5B.

Given the discovery of these additional errors that were made in terms of the assembly of two other figures in the above paper, the Editor of *Oncology Reports* has decided that this article should now be retracted from the Journal on account of a lack of confidence in the originally presented data. The authors were asked for an explanation to account for these concerns, but the Editorial Office did not receive a reply. The Editor apologizes to the readership for any inconvenience caused.

